# Development, Validation, and Comparison of Image-Based, Clinical Feature-Based and Fusion Artificial Intelligence Diagnostic Models in Differentiating Benign and Malignant Pulmonary Ground-Glass Nodules

**DOI:** 10.3389/fonc.2022.892890

**Published:** 2022-06-07

**Authors:** Xiang Wang, Man Gao, Jicai Xie, Yanfang Deng, Wenting Tu, Hua Yang, Shuang Liang, Panlong Xu, Mingzi Zhang, Yang Lu, ChiCheng Fu, Qiong Li, Li Fan, Shiyuan Liu

**Affiliations:** ^1^ Department of Radiology, Changzheng Hospital, Navy Medical University, Shanghai, China; ^2^ Department of Radiology, The Second People’s Hospital of Yuhuan, Yuhuan, China; ^3^ Department of Radiology, Longyan First Affiliated Hospital of Fujian Medical University, Fujian, China; ^4^ Department of Research, Shanghai Aitrox Technology Corporation Limited, Shanghai, China; ^5^ Department of Radiology, Sun Yat-sen University Cancer Center (SYSUCC), Guangzhou, China

**Keywords:** ground-glass nodule, computed tomography, differential diagnosis, computer-aided diagnosis, artificial intelligence

## Abstract

**Objective:**

This study aimed to develop effective artificial intelligence (AI) diagnostic models based on CT images of pulmonary nodules only, on descriptional and quantitative clinical or image features, or on a combination of both to differentiate benign and malignant ground-glass nodules (GGNs) to assist in the determination of surgical intervention.

**Methods:**

Our study included a total of 867 nodules (benign nodules: 112; malignant nodules: 755) with postoperative pathological diagnoses from two centers. For the diagnostic models to discriminate between benign and malignant GGNs, we adopted three different artificial intelligence (AI) approaches: a) an image-based deep learning approach to build a deep neural network (DNN); b) a clinical feature-based machine learning approach based on the clinical and image features of nodules; c) a fusion diagnostic model integrating the original images and the clinical and image features. The performance of the models was evaluated on an internal test dataset (the “Changzheng Dataset”) and an independent test dataset collected from an external institute (the “Longyan Dataset”). In addition, the performance of automatic diagnostic models was compared with that of manual evaluations by two radiologists on the ‘Longyan dataset’.

**Results:**

The image-based deep learning model achieved an appealing diagnostic performance, yielding AUC values of 0.75 (95% confidence interval [CI]: 0.62, 0.89) and 0.76 (95% CI: 0.61, 0.90), respectively, on both the Changzheng and Longyan datasets. The clinical feature-based machine learning model performed well on the Changzheng dataset (AUC, 0.80 [95% CI: 0.64, 0.96]), whereas it performed poorly on the Longyan dataset (AUC, 0.62 [95% CI: 0.42, 0.83]). The fusion diagnostic model achieved the best performance on both the Changzheng dataset (AUC, 0.82 [95% CI: 0.71-0.93]) and the Longyan dataset (AUC, 0.83 [95% CI: 0.70-0.96]), and it achieved a better specificity (0.69) than the radiologists (0.33-0.44) on the Longyan dataset.

**Conclusion:**

The deep learning models, including both the image-based deep learning model and the fusion model, have the ability to assist radiologists in differentiating between benign and malignant nodules for the precise management of patients with GGNs.

## Introduction

Lung cancer remains the leading cause of global cancer deaths, especially in China (1, 2). Since the low-dose multi-detector spiral CT was introduced to lung cancer screening, the number of detected ground-glass nodules (GGNs) has dramatically increased (3, 4). In contrast to solid nodules, GGNs have a higher malignancy rate (5–7), even though benign GGNs were also frequently reported in postoperative pathologies, such as focal pneumonia, organizing pneumonia, focal fibrosis, lipoid pneumonia, pulmonary hemorrhage (8, 9). Although early detection and the subsequent resection of malignant GGNs may improve the prognosis of patients, it has a hard time differentiating between benign and malignant nodules for radiologists. Moreover, the discrimination between benign and malignant nodules is of critical importance to an appropriate and consistent treatment strategy for patients suspected of early-stage lung cancer, which has now become a crucial clinical issue. Accurate diagnosis plays an essential role in GGNs management and provides a foundation for choosing appropriate treatment and predicting prognosis.

However, due to imaging resemblance, it is incredibly challenging to differentiate malignant GGNs from their benign counterparts. The morphologic characteristics of malignant pulmonary nodules are similar to those of benign pulmonary nodules (10, 11). Diagnosis of GGNs has remained a challenge with dedicated CT, FDG-PET/CT, or even image-guided percutaneous biopsy, However, these technological advances have the potential to define a new era in the evaluation of GGNs. PET-CT is a functional imaging method demonstrating differences in the glucose metabolism of tissues. As infection and inflammatory lesions are also hypermetabolic, the efficacy of PET-CT to differentiate benign and malignant lesions has been restricted (12). Despite advances in nonsurgical biopsy techniques, unnecessary surgical resections of low-risk nodules or benign nodules remain common. Thus, accurate discrimination between benign and malignant GGNs could never be overemphasized in the process of improving patient management.

There is no single robust method for differentiating benign GGNs from malignant ones. Deep learning technologies such as convolutional neural network (CNN) demonstrate outstanding potential in extracting comprehensive features from extensive sets of complex data (13–15). In addition, those technologies have been successfully applied to the diagnosis of disease, the evaluation of prognosis, and the prediction of pathological response in Non-small Cell Lung Carcinoma (NSCLC) (16–18). Therefore, it is expected to become a simple, convenient, reproducible, and noninvasive method to differentiate between malignant and benign nodules. Many studies reported deep learning models which had achieved unprecedented success in differentiating malignant and benign pulmonary nodules. However, most of them were based mainly on public datasets without pathological diagnoses for the included nodules as gold standards (19–21). Besides, most studies were based on solid nodules, and only a few were on benign or malignant GGNs.

This study aimed to develop diagnostic models based on CT image patches of GGN, clinical characteristics of patients and image features, or a combination of both, in the task of differentiating benign and malignant ground-glass nodules (GGNs) with pathological diagnoses, and to compare the diagnostic performance of these models against manual evaluation by radiologists.

## Methods

### Study Population

**Figure 1:**
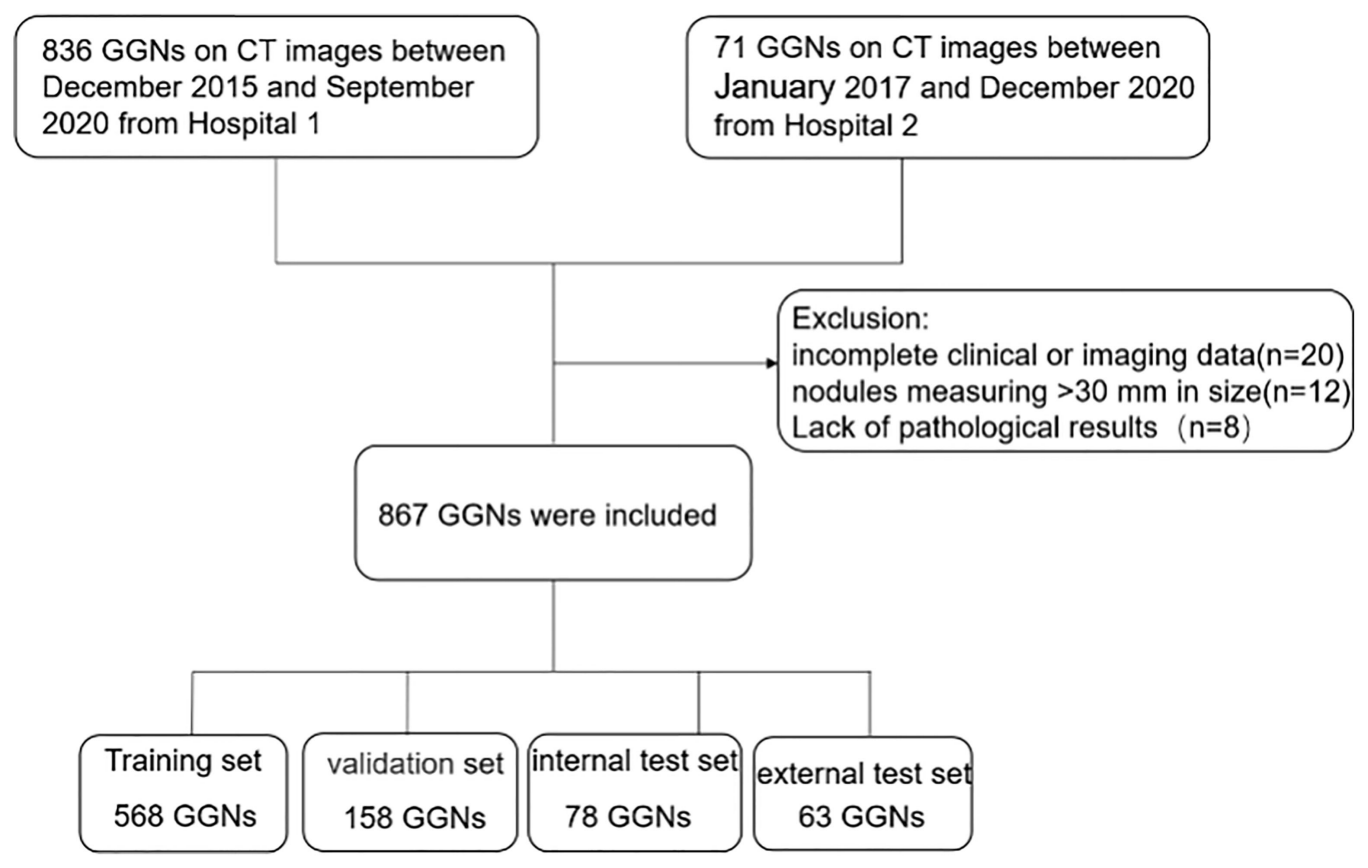
Flowchart of study population. CT, computed tomography; GGN, ground-glass nodule.

The institutional review board of the local hospitals approved this retrospective study (Changzheng Hospital, No.2018SL028), and the written informed consent from patients was waived due to its retrospective nature. A search using the keywords “GGN”, “ground-glass opacity”, “part-solid nodule”, and “ground-glass” in CT reports was performed to screen out patients with GGNs admitted to Changzheng hospital in the period from December 2015 to September 2020 and Longyan First hospital in the period from January 2017 to December 2020. The inclusion criteria were: (a) nodules with the pathological diagnosis made on specimens obtained by CT-guided transthoracic needle biopsy, transbronchial biopsy, video-assisted thoracoscopic surgery, or surgical resection; (b) GGNs measuring <30 mm in size; and (c) images with a slice thickness of 1-mm or 0.625-mm. The exclusion criteria were: (a) incomplete clinical or imaging data; (b) GGNs described in histopathological reports not identifiable on CT images; (c) image of insufficient quality (e.g. artifacts in CT images). The patient inclusion procedure is shown in **Figure 1**. We collected only the latest CT images prior to their surgery.

A total of 867 nodules (benign: 112; malignant: 755) from the two independent datasets were included in our study, of which 804 GGNs (the ‘Changzheng Dataset’, benign nodules: 102; adenocarcinoma *in situ* (AIS) nodules: 175; minimally invasive adenocarcinoma (MIA) nodules: 192; invasive adenocarcinoma (IA) nodules: 335) were collected from Changzheng hospital to train and validate the models. A dataset consisting of 63 GGNs (benign nodules: 10; AIS nodules: 5; MIA nodules: 24; IA nodules: 24) collected from Longyan First Hospital was established as an external test set (the ‘Longyan Dataset’).

Patient images in the Changzheng dataset were obtained using five different CT scanners (TOSHIBA Aquilion, two Philips Ingenuity scanners, General Electric LightSpeed VCT, and Philips iCT256). CT images in the Longyan dataset were obtained using the Philips iCT256. All CT images were acquired in the supine position at full inspiration. Scan coverage was from the adrenal gland to the thoracic inlet. Scanning parameters were 120 kV, 50-150 mA, image matrix 512 × 512 pixels, and 0.5-second scanning duration. Continuous images were reconstructed with a thickness of 0.625-1mm. All images were exported in DICOM format.

### Nodule Labeling

All CT morphology characteristics were reviewed by two thoracic radiologists (W.X, and l.Q, respectively, with five and ten years of experience in chest CT) who were blinded to the pathological results. Based on the presence of a solid component, nodules were classified into two groups: the pure ground-glass nodule (pGGN) group and the mixed ground-glass nodule (mGGN) group. On high-resolution CT images, the pGGN was defined as an area of hazy increased lung attenuation with distinct margins of underlying vessels and bronchial walls; the mGGN was characterized as nodules with both ground-glass and solid components.

Tumor segmentation was performed using an in-house software tool Prolego (Image Processing System, Aitrox Technology Corporation Limited, Shanghai, China). For each tumor, regions of interest (ROI) on the entire three-dimensional range of the axial CT images covering the tumor, was first drawn by Prolego. The methods for evaluating segmentation results had previously been validated (22). The maximum dimension on axial CT images was measured and recorded by the two radiologists who independently made qualitative (attenuation, lobulation, spiculation, air bronchogram, pleural indentation, vacuole sign and nodule-lung interface) and quantitative (the maximum diameters of the lesions in the transverse plane) assessments in CT images. Three clinical parameters (age, sex, smoking history [never- smoker, current and former smoker]) were disclosed to observers. The basic characteristics of the two independent datasets are summarized in **Table 1**.

**Table 1:** Characteristics of the GGNs in two datasets.

Characteristic	Changzheng Dataset N=743 (804 Nodules)	Longyan Dataset N=61 (63 Nodules)
**Benign**	102(12.7)	10(15.9)
**Adenocarcinoma stage**		
**AIS**	175(21.8)	5(7.9)
**MIA**	192(23.9)	24(38.1)
**IA**	335(41.6)	24(38.1)
Age*	55[48,63]	53[42,61]
Sex*		
**Male**	266(35.8)	22(36.1)
**Female**	477(64.2)	39(63.9)
**Location**		
**RUL**	303(37.6)	15(23.8)
**RML**	56(7.0)	8(12.7)
**RLL**	147(18.3)	14(22.2)
**LUL**	200(24.9)	20(31.8)
**LLL**	98(12.2)	6(9.5)
Smoker*		
**Yes**	119(16.0)	10(16.4)
**No**	624(84.0)	51(83.6)
**Maximal Diameter**	14.0[10.5,18.0]	10.0[8,16.75]
**Diameter Range**		
**(0, 10]**	201(25.0)	34(54.0)
**(10, 20]**	466(58.0)	23(36.5)
**(20, 30]**	137(17.0)	6(9.5)
**pGGN**	468(58.2)	35(55.6)
**mGGN**	336(41.8)	28(44.4)

The characteristics with * are counted on patient level, others are counted on nodule level. AIS, adenocarcinoma in situ; MIA, minimally invasive adenocarcinoma; IAC, invasive adenocarcinoma; RUL, right upper lobe; RML, right middle lobe; RLL, right lower lobe; LUL, left upper lobe; LLL, left lower lobe; pGGN, pure ground glass nodule; mGGN, mixed ground glass nodule.

### Pathological Diagnosis

The pathological subtypes of all malignant GGNs were categorized according to the 2015 pulmonary adenocarcinoma classification (23). All pathological specimens of each case were confirmed by at least two experienced pathologists and benign cases were histopathological confirmed with hemorrhage, chronic inflammation, and focal interstitial fibrosis. The consensus was reached by mutual discussion or consultation with a third pathologist whenever there was a disagreement.

### Diagnostic Models

#### Image-Based Deep Learning Diagnostic Model

##### Data Pre-Processing

The window level of all included CT images was reset to -200 HU, and the window width was reset to 800 HU. All voxels were subsequently normalized to the range of [0,1]. An image patch with the size of 32x32x32 pixels was cut around the nodule position annotated by radiologists as the input of the model.

Before training, we performed data augmentation for benign cases to rectify the classification bias due to the imbalance in sample size. The data augmentation included an image shifting procedure, where the image patches were randomly shifted within five pixels on the x- and y-axes, and an image rotation procedure, where the patches were randomly rotated 90°, 180°, or 270°on x-y plan, x-z plan, and y-z plan. After data augmentation for benign cases, the ratio of benign and malignant nodules was approximately 1:1.

#### Construction of the Neural Network

**Figure 2:**
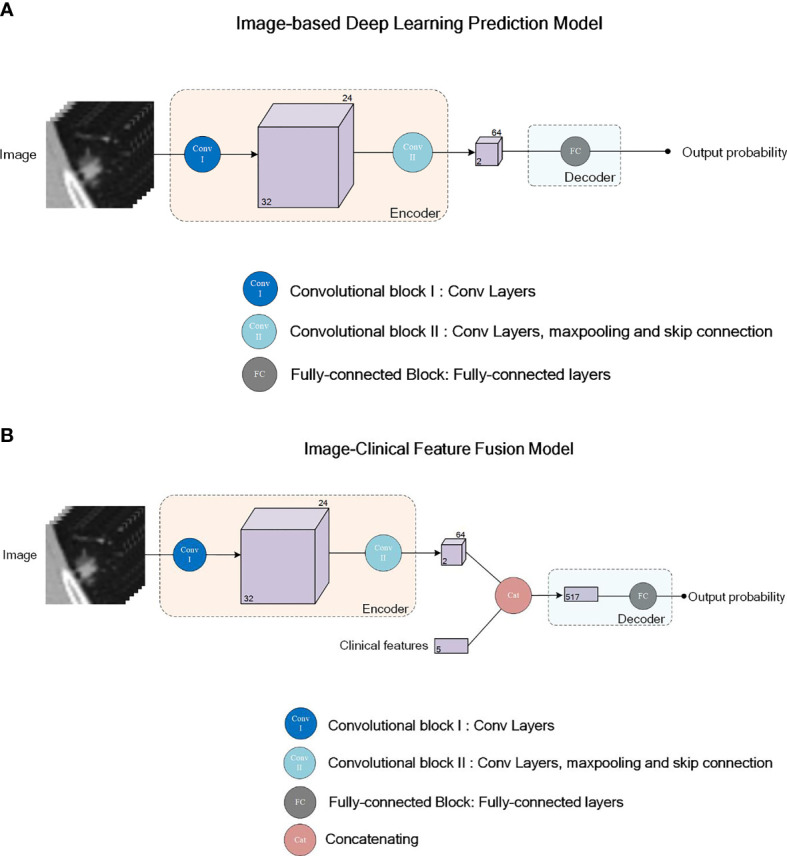
Network structure illustration for the deep learning models IBTL **(A)** and FPM **(B)**. Convolutional block I (Conv I) consists of 2 convolutional layers. Convolutional block II (Conv II) consists of 4 Resnet sub-blocks and maxpooling layers after each Resnet sub-block. First two of the Resnet sub-block consists of 4 convolutional layers each, while the last two Resnet sub-blocks consists of 6 convolutional layers each. Skipping connection is adopted in all 4 Resnet-sub blocks. Fully-connected block (FC) consists of 3 fully-connected layers.

We used the DenseNet (24), which has been successfully applied in many medical image classification tasks, as the network backbone for our image-based deep learning diagnostic model. The input was an image patch covering the whole nodule in the size of 32x32x32 pixels. The output was a single value in the range of [0,1], indicating the probability of malignancy of the nodule. As shown in **Figure 2A**, our deep learning diagnostic model consisted of two convolutional blocks (Conv I, II) and a fully-connected block (FC). The model included an encoder network and a decoder network. The encoder network, consisting of Conv I and II, was used to extract image features from the input image patches, followed by the FC block’s decoder network, which was used to calculate the classification probabilities according to features extracted by the encoder along with the sigmoid function. Two models with the same network architecture were trained with different strategies: the Image-Based Deep Learning model without Transfer Learning trained *de novo*, namely IBDL-nonTL (for Image-Based Deep Learning model – non Transfer Learning); and the Image-Based Deep Learning model with Transfer Learning loaded with parameters pre-trained with ImageNet, namely IBDL-TL (for Image-Based Deep Learning model –Transfer Learning). We used the cross entropy as the loss function in the model training process. Adam optimizer with an initial learning rate of 5x10^-5^ was used to optimize the weights for IBDL-TL. The same optimizer with an initial learning rate of 1x10^-3^ was used for IBDL-nonTL.

#### Clinical Feature-Based Diagnostic Model

A logistic regression model based on clinical features (age, sex, smoking history) and image features (maximum diameters, attenuation, lobulation, spiculation, air bronchogram, pleural indentation, vacuole sign and nodule-lung interface) was constructed to diagnose the malignancy of pulmonary nodules, namely CFBLR for Clinical Feature-Based Linear Regression Model. Patients were divided into the benign and malignant groups according to the pathological diagnoses. The difference in the distribution of each feature between the two groups was statistically analyzed. Only features with statistically significant differences between the two groups were fed to the diagnostic model as input.

The logistic regression model was constructed, trained, and assessed using the Scikit-learn library (25) (Version 1.0) on the Python platform (Version 3.6.8, Python Software Foundation, USA). All hyper-parameters of the linear regression model were set as default.

#### Image-Clinical Feature Fusion Model

For a more accurate determination of pulmonary nodule malignancy, we constructed a fusion model upon the image-based deep learning models, integrating the original CT images, clinical features of the patients, and manually extracted image features. As shown in **Figure 2B**, the clinical features and manually extracted image features were associated with the high-dimensional image features extracted from CT images by the encoder network. The clinical features, the manually extracted image features, and the encoder extracted image features, were passed to the decoder together to evaluate based on fusion information. The structure of the ultimate decoder was the same as that for the image-based diagnostic model, except for having employed the extra clinical features and manually extracted image features as the inputs.

Similar to the image-based deep learning models, two fusion models adopting the same network architecture were trained with different strategies: the Fusion Prediction Model without Transfer Learning (FPM-nonTL, for Fusion Prediction Model – non Transfer Learning) was trained *de novo*; and the Fusion Prediction Model with Transfer Learning (FPM-TL, for Fusion Prediction Model – Transfer Learning) was loaded with parameters pre-trained with ImageNet.

#### Model Evaluation and Statistical Analysis

The performance of our classification models was evaluated by the receiver operating characteristic (ROC) curve and the area under the ROC curve (AUC). We examined the models’ sensitivity, specificity, accuracy, positive predictive value (PPV), and negative predictive value (NPV) at the probability threshold of 0.5.

The ROC curves and the metrics were obtained using the R language platform (Version 4.0.0, R Foundation for Statistical Computing, Vienna, Austria). Differences in the diagnostic performance between the diagnostic models and real-world radiologists were compared on the Longyan dataset in terms of sensitivity, specificity, accuracy, PPV and NPV.

Comparison of patient demographics between different groups was performed using MedCalc (Version 18.2.1, MedCalc Software Ltd, Belgium). A mono-factor analysis including statistically significant clinical features was performed using Python (Version 3.6.8, Python Software Foundation, USA). A p-value less than 0.05 indicated statistical significance. To compare the differences in categorical variables (sex, nodule-lung interface, pleural indentation, specular sign, smoking history, nodule attenuation, lobulation, vacuole sign, and air bronchogram) between groups, Chi-square tests were applied. For continuous variables, we used the Shapiro-Wilk test to check for normality before a Mann-Whitney U Test was used for non-normally distributed data or an independent two-sided t-test for normally distributed data. To compare the performance of classification models on the same test dataset, DeLong Test was applied for the ROC curves.

## Results

A total of 804 patients (mean age, 55 ± 11 years; range, 20–87 years) were included in this study, including 516 (64.17%) women (mean age, 54 ± 11 years; range,25–87 years) and 288 (35.82%) men (mean age, 55 ± 11 years; range, 20–79 years). A hundred and twenty-nine (16.04%) patients had a smoking history. Demographic characteristics and CT morphological characteristics of the 867 GGNs (benign nodules: 112; malignant nodules: 755) are summarized in **Table 2**. The maximum diameters of malignant GGNs were significantly more than the benign GGNs (P<0.001), and malignant GGNs are more likely to have a well-defined border (P<0.001).

**Table 2:** Statistical analysis of clinical features between the malignant and benign cases in the Changzheng Dataset.

Feature	Benign cases	Malignant cases	p-value
**Age**	55[46,61]	55[48,63]	0.45
**Sex**			
**Male**	49	243	0.01*
**Female**	53	459	
**Nodule-lung Interface**			
**Unclear**	34	123	<0.001*
**Clear**	68	579	
**Pleural Indentation**			
**With**	7	90	0.12
**Without**	95	612	
**Spicular Sign**			
**With**	2	29	0.43
**Without**	100	673	
**Smoking History**			
**Yes**	8	120	0.017*
**No**	94	582	
**Maximal diameter (mm)**	12[8,15]	14[11,19]	<0.001*
**Attenuation**			
**pGGN**	51	417	0.09
**mGGN**	51	285	
**Spiculation Sign**			
**With**	19	157	0.47
**Without**	83	545	
**Vacuole Sign**			
**With**	2	12	
**Without**	100	690	0.82
**Air Bronchogram**			
**With**	13	32	
**Without**	89	670	0.002*

pGGN: pure ground glass nodule, mGGN: mixed ground glass nodule.

*p value < 0.05

**Figure 3:**
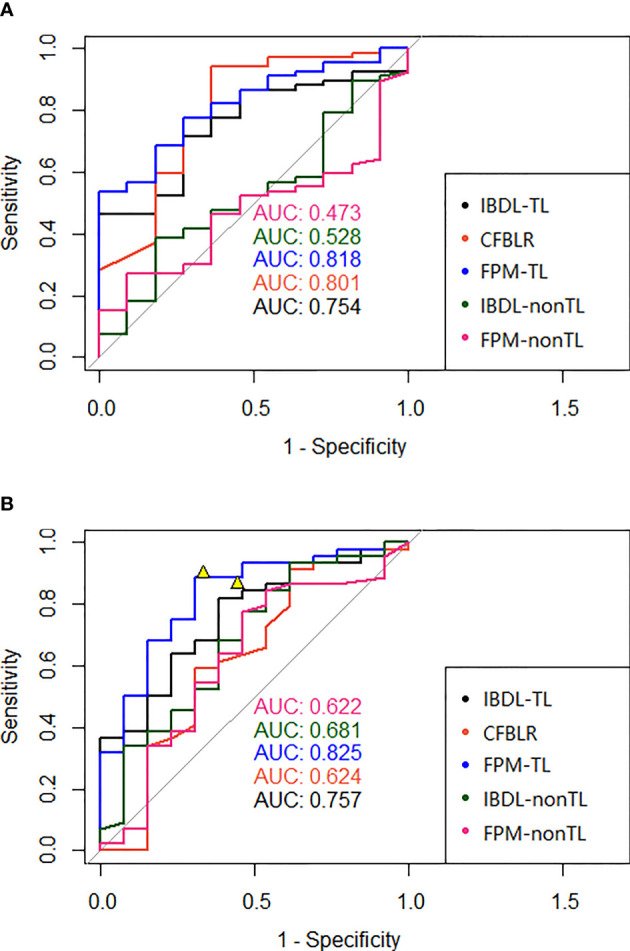
**(A)** The ROC of each model (clinical model, two image feature models with or without transfer learning, and two fusion models with or without transfer learning) in the test data set of our hospital was presented in **(A)**; **(B)** The ROC of each model (clinical model, two image feature models with or without transfer learning, and two fusion models with or without transfer learning) of the independent test data set in the external hospital was presented in **(B)**. The figures also showed two representative points of the interpretation doctors. AUC, Area under the ROC curve; IBDL-TL, Image-based Deep Learning (transfer learning) model; CFBLR, clinical feature based regression; FPM-TL, fusion prediction model (transfer learning); IBDL-nonTL, Image-based Deep Learning (non-transfer learning) model; FPM-nonTL, fusion prediction model (non-transfer learning).

IBDL-TL achieved a similar performance on the Changzheng and Longyan dataset, with AUC values of 0.75 (95% CI: 0.62, 0.89) and 0.76 (95% CI: 0.61, 0.90), respectively. (**Figure 3** and **Table 3**) Corresponding to the threshold of malignancy possibility at 0.5, the model achieved sensitivities of 0.61 and 0.68, specificities of 0.73 and 0.62, and accuracies of 0.63 and 0.67, for the two datasets, respectively. The PPVs and NPVs of the IBDL-TL model are also shown in **Table 3**. In contrast, IBDL-nonTL yielded a much lower performance on the two test datasets, respectively, with AUC values of 0.53 (95% CI: 0.35, 0.71) and 0.68 (95% CI: 0.50, 0.86), sensitivities of 0.33 and 0.82, and specificities of 0.82 and 0.46, respectively, for the two test datasets. Comparison between AUCs of the IBDL-TL and IBDL-nonTL on the Changzheng test dataset, indicates that the performance of IBDL-TL is better than IBDL-nonTL with statistical significance. (p=0.042)

**Table 3:** Summary of diagnostic indicators for each model and two clinicians (Our hospital, external hospital: AUC, sensitivity, specificity, accuracy, PPV and NPV).

	Changzheng Test Dataset							Longyan Test Dataset						
	AUC	P-value*	sensitivity	specificity	accuracy	PPV	NPV	AUC	P-value*	sensitivity	specificity	accuracy	PPV	NPV
**IBDL‐TL**	0.75 (0.62‐0.89)	0.267	0.61	0.73	0.63	0.93	0.24	0.76 (0.61‐0.90)	0.290	0.68	0.62	0.67	0.86	0.36
**IBDL‐nonTL**	0.53 (0.35‐0.71)	0.003	0.33	0.82	0.40	0.92	0.17	0.68 (0.50‐0.86)	0.089	0.82	0.46	0.74	0.84	0.43
**CFBLR**	0.80 (0.64‐0.96)	0.864	0.82	0.64	0.79	0.93	0.37	0.62 (0.42‐0.83)	0.018	0.82	0.38	0.72	0.82	0.38
**FPM‐TL**	0.82 (0.71‐0.93)	----	0.79	0.64	0.77	0.93	0.33	0.83 (0.70‐0.96)	----	0.77	0.69	0.75	0.89	0.47
**FPM‐nonTL**	0.47 (0.32‐0.63)	0.0001	0.16	0.91	0.27	0.92	0.15	0.62 (0.43‐0.81)	0.068	0.16	0.85	0.32	0.78	0.23
**Radiologist R1**	NA	NA	NA	NA	NA	NA	NA	NA	NA	0.87	0.44	0.81	0.91	0.33
**Radiologist R2**	NA	NA	NA	NA	NA	NA	NA	NA	NA	0.9	0.33	0.83	0.9	0.33

*For the DeLong Test between the corresponding model performance and that of FPM-TL on the same test dataset. NA, not applicable.

Compared with the IBDL-TL, CFBLR achieved better performance on the Changzheng dataset than on the Longyan dataset (**Figure 3** and **Table 3**), with AUC values of 0.80 (95% CI: 0.64, 0.96) and 0.62 (95% CI: 0.42, 0.83), sensitivities of 0.82 and 0.82, specificities of 0.64 and 0.38, and accuracies of 0.79 and 0.72 for the two test datasets. The corresponding PPV and NPV values are shown in **Table 3**. The five most important features of the CFBLR model and their weights are listed in **Table 4**.

**Table 4:** The five most important features of the CFBLR model and their weights.

Feature	Weight
**Sex**	-0.993
**Nodule-lung Interface**	-1.277
**Smoking History**	2
**Maximal Diameter**	0.12
**Air Bronchogram**	-1.354

FPM-TL achieved the best performance out of our models, with AUC values of 0.82 (95% CI: 0.71, 0.93) and 0.83 (95% CI: 0.70, 0.96), sensitivities of 0.79 and 0.77, specificities of 0.64 and 0.69, and accuracies of 0.77 and 0.75, respectively, for the Changzheng and Longyan datasets (**Figure 3** and **Table 3**). While DeLong tests showed no statistical significance between FPM-TL and IBDL-TL or CFBLR on Changzheng test dataset (all p>0.05), the performance of FPM-TL was better than CFBLR on the Longyan test dataset with statistical significance (p=0.018). Compared with FPM-TL, the performance of FPM-nonTL was inferior (p=0.0001), with AUC values of 0.47 (95% CI: 0.32, 0.63) and 0.62 (95% CI: 0.43, 0.81), sensitivities of 0.16 and 0.16, and specificities of 0.91 and 0.85 on the Changzheng and Longyan datasets, respectively.

In our study, two radiologists evaluated benign and malignant GGNs independently by the Longyan dataset. The results showed that sensitivities achieved by radiologists ranged from 0.87 to 0.9. The overall accuracy of radiologists ranged from 0.81 to 0.83 (**Figure 3** and **Table 3**).

## Discussion

Our results suggested that the IBDL-TL model could effectively distinguish benign and malignant GGNs on both the Changzheng dataset and the Longyan dataset. This reflected a great generalizability of the IBDL-TL model since tests on the two independent datasets suggested similar AUC values (0.75 and 0.76). Compared with the IBDL-TL model, although the CFBLR model (trained upon the manually extracted clinical features) achieved a higher AUC of 0.80 on the Changzheng dataset, its performance on the Longyan dataset was much lower (AUC = 0.62), suggesting a lack of cross-center generalizability. Therefore, this could be a severe problem if it was applied to real radiological practice. In addition, our results revealed that a proper fusion of image information, clinical features and radiological features manually extracted from CT images could contribute to a higher diagnostic efficacy on both the Changzheng and Longyan datasets (AUC values, 0.82 and 0.83). It is noteworthy that the fusion model showed an excellent generalizability while its AUC values were the highest among all models on both test datasets, which indicated promising potential for it to be applied in clinical practice.

The malignancy of pulmonary nodules may be distinguished based on patients’ clinical information (sex, smoking history, etc.) and accurate CT imaging characteristics. It is controversial whether preoperative CT morphological features of chronic inflammatory could differentiate between benign and malignant nodules. In our CFBLR model, a larger diameter of the nodule indicated greater probability of malignancy. Some studies (26, 27) have indicated that the malignancy is extremely low (<1%) for nodules less than 5 mm and 64%-82% for nodules larger than 20 mm. Besides, smoking is a risk factor for lung cancer. The smoking rate of patients in the malignant cohort of the Changzheng dataset was 20.6%, which was much lower than that of other studies (28, 29). However, we found that female was closely associated with malignant pulmonary nodules (p = 0.01), which was in line with previous studies (30–32). The results of our studies showed that a well-defined border was also significantly associated with malignant GGNs, inconsistent with previous *in vivo* studies (33). Besides, our results on air branchogram were in conflict with previous studies (34). We demonstrated the presence of more air bronchogram in benign GGNs (**Table 2**). This may be related to our case selection bias and the uneven number of benign and malignant cases. Besides, our benign cases were mainly proved by surgery, and the signs were usually atypical. This also showed the limitations of distinguishing between the benign and malignant by the CT morphological features. The model will be optimized as the sample size increases later.

This study illustrated that the deep learning model could accurately differentiate between benign and malignant GGNs, and may help reduce misdiagnosis or overtreatment of GGNs. Hu et al. (35) classified 89 GGNs as benign or malignant using 396 quantitative texture features, achieving an AUC of 72.9% in the validation dataset. Gong et al. (36) established a classification method based on the fusion of radiomics features and deep learning features and achieved an AUC of 0.73 for the test dataset. Our study demonstrated that the IBDL-TL and FPM-TL models performed well on both the Changzheng and Longyan datasets. Furthermore, a comparison of the diagnostic performance between the IBDL-TL and FPM-TL models and radiologists showed that although the AI models have slightly lower sensitivities (ranging from 0.68 to 0.77, compared to sensitivities ranging from 0.87 to 0.9 of radiologists), both the IBDL-TL and FPM-TL models have higher specificities (ranging from 0.62 to 0.69) than radiologists (ranging from 0.33 to o.44), suggesting the potential of the models for reducing false positives in future clinical applications.

In the technical aspect, the critical issues in the development of effective diagnostic models for distinguishing between malignant and benign GGNs include 1) how to deal with the severe imbalance between positive and the negative samples; 2) how to compensate for the lack of enough training data; 3) how to effectively fuse the clinical features and the image information to construct a diagnostic model. The imbalance of samples between classes made it difficult for the model to learn negative features. In addition, models trained with unbalanced training datasets would tend to make a false positive diagnosis. To solve this problem, we used a data augmentation strategy, applied by many studies in medical-image-based classification tasks (14, 37). The negative image patches were augmented by image shifting and rotating. Thus, the model could learn features from more negative image patches. Besides, during the training process, we randomly picked 32 samples from positive image patches and 32 samples from augmented negative samples to balance the positive and negative samples in each training batch. In this way, the bias of the model could be prevented. The lack of training samples made the model overfitting on the training dataset and underperforming on the test dataset. To deal with this problem, we applied a transfer learning strategy, where network weights pre-trained with ImageNet were loaded into the encoder. As proved by many studies (38–40), the transfer learning strategy can effectively train a well performing classification model with relatively small sample size.

Furthermore, a meager learning rate of 1x10-5 was used to prevent overfitting. For an efficient fusion of clinical features and manually extracted image features with CT images, relatively abstract clinical features and manually extracted image features were fused with the information from CT image patches only after the high-dimensional feature information had been extracted from the raw image patches. This was because, in theory, the information extracted by the encoder and the manually extracted information was of similar abstract levels.

In addition, two radiologists of different seniority reviewed the test dataset in the meantime (**Table 3**). The results showed that the radiologists achieved sensitivities of 0.87 and 0.9 and accuracies of 0.81 and 0.83, which suggested that our proposed models outperformed the performance of the two radiologists. The current findings indicated that the deep learning-based pulmonary nodule assessment model could increase diagnostic accuracy and radiologists’ productivity. In this study, we built a database and another independent test database upon GGNs with pathological diagnoses to train and test the classification model. Unlike Computer-aided diagnosis (CAD) schemes with publicly available datasets lacking histopathologically confirmed results, our proposed models were trained and tested with histopathology confirmed nodules. In addition, clinical features and CT features were intelligently integrated into the deep learning models. The evaluation capability of our method was further enhanced compared with methods built with a single deep learning model or clinical features.

## Limitations

Our study had several limitations. First, as a retrospective analysis, almost all patients in this study had pathology, suggesting all these cases were suspected to be malignant by clinicians. Thus, the selection bias was unavoidable. Second, we included only pathologically diagnosed GGNs after surgery, therefore the number of benign GGNs in our study was relatively small. Third, the sample size of the external test set was relatively small; In this case, performance of those developed models need to be verified on larger datasets collected from multiple external centers.

## Conclusions

We developed AI diagnostic models to classify GGNs on CT images. Our findings suggested that the deep learning approaches achieved an excellent performance in classifying GGNs nodules, compared to the performance of radiologists. This study provided scientific evidence that deep learning methods may improve the classification performance of benign and malignant nodules. These models may provide a non-invasive, fast, low-cost, and reproducible method to accurately differentiate between benign and malignant GGNs, which would tremendously benefit the management of patients with GGNs.

## Data Availability Statement

The original contributions presented in the study are included in the article/Supplementary Material. Further inquiries can be directed to the corresponding authors.

## Ethics Statement

Our study was approved by the ethics committee of Changzheng Hospital for retrospective analysis and did not require informed consent (No.2018SL028). The patients/participants provided their written informed consent to participate in this study.

## Author Contributions

XW, MG and JX contributed equally to this work. XW and MG, YD and WT conducted data acquisition, collection and interpretation. XW made major contributions in manuscript drafting. CF and YL made important contributions to the model designing and building. HY made of image preprocessing and model interpretation. PX contributed to the building of clinical feature-based model. MZ made contribution to the manuscript editing. JX made statistical analysis. LF made contribute in the data acquisition and annotation. SL assisted in building the model. QL made contribute in annotation. SYL supervised the model designing, building and manuscript editing. SYL had final responsibility for the decision to submit for publication.

## Funding

This study was funded by the National Natural Science Foundation of China (Grant number 82001812), Pyramid Talent Project of Shanghai Changzheng Hospital, Shanghai Municipal Commission of Health and Family Planning Program (grant number 20184Y0037, Shanghai Sailing Program (grant number 20YF1449000), Shanghai Municipal Commission of Health and Family Planning Program (grant number 2018ZHYL0101), The key project of the National Natural Science Foundation of China (grant number 81930049).

## Conflict of Interest

Authors HY, SL, PX, MZ, YL, and CF were employed by Aitrox Technology Corporation Limited.

The remaining authors declare that the research was conducted in the absence of any commercial or financial relationships that could be construed as a potential conflict of interest.

## Publisher’s Note

All claims expressed in this article are solely those of the authors and do not necessarily represent those of their affiliated organizations, or those of the publisher, the editors and the reviewers. Any product that may be evaluated in this article, or claim that may be made by its manufacturer, is not guaranteed or endorsed by the publisher.

## References

[1] Chen W Zheng R Baade PD Zhang S He J. Cancer Statistics in China, 2015 Chen et al-2016. CA A Cancer J Clin (2016) 66:115–32. doi: 10.3322/caac.21338 26808342

[2] Sung H Ferlay J Siegel RL Laversanne M Soerjomataram I Jemal A. Global Cancer Statistics 2020: GLOBOCAN Estimates of Incidence and Mortality Worldwide for 36 Cancers in 185 Countries. CA Cancer J Clin (2021) 71(3):209–249. doi: 10.3322/caac.21660 33538338

[3] Henschke CI Yankelevitz DF Mirtcheva R McGuinness G McCauley D Miettinen OS. CT Screening for Lung Cancer: Frequency and Significance of Part-Solid and Nonsolid Nodules. AJR Am J Roentgenol (2002) 178(5):1053–7. doi: 10.2214/ajr.178.5.1781053 11959700

[4] Wang YH Chen CF Lin YK Chiang C Tzao C Yen Y. Predicting Malignancy: Subsolid Nodules Detected on LDCT in a Surgical Cohort of East Asian Patients. J Thorac Dis (2020) 12(8):4315–26. doi: 10.21037/jtd-20-659 PMC7475597 32944344

[5] Cai J Xu D Liu S Cham MD. The Added Value of Computer-Aided Detection of Small Pulmonary Nodules and Missed Lung Cancers. J Thorac Imaging (2018) 33(6):390–5. doi: 10.1097/RTI.0000000000000362 30239461

[6] Nakata M Saeki H Takata I Segawa Y Mogami H Mandai K. Focal Ground-Glass Opacity Detected by Low-Dose Helical CT. Chest (2002) 121(5):1464–7. doi: 10.1378/chest.121.5.1464 12006429

[7] Kim HY Shim YM Lee KS Han J Yi CA Kim YK. Persistent Pulmonary Nodular Ground-Glass Opacity at Thin-Section CT: Histopathologic Comparisons. Radiology (2007) 245(1):267–75. doi: 10.1148/radiol.2451061682 17885195

[8] Fan L Liu SY Li QC Yu H Xiao XS. Multidetector CT Features of Pulmonary Focal Ground-Glass Opacity: Differences Between Benign and Malignant. Br J Radiol (1015) 2012:897–904:85. doi: 10.1259/bjr/33150223 PMC3474071 22128130

[9] Park CM Goo JM Lee HJ Lee CH Chun EJ Im JG. Nodular Ground-Glass Opacity at Thin-Section CT: Histologic Correlation and Evaluation of Change at Follow-Up. Radiographics (2007) 27(2):391–408. doi: 10.1148/rg.272065061 17374860

[10] Ohno Y Nishio M Koyama H Miura S Yoshikawa T Matsumoto S. Dynamic Contrast-Enhanced CT and MRI for Pulmonary Nodule Assessment. AJR Am J Roentgenol (2014) 202(3):515–29. doi: 10.2214/AJR.13.11888 24555587

[11] Kim TJ Kim CH Lee HY Chung MJ Lee KSJ. Management of Incidental Pulmonary Nodules: Current Strategies and Future Perspectives. Expert Rev Respir Med (2019) 14(3):173–94. doi: 10.1080/17476348.2020.1697853 31762330

[12] Nomori H Watanabe K Ohtsuka T Naruke T Suemasu K Uno K. Evaluation of F-18 Fluorodeoxyglucose (FDG) PET Scanning for Pulmonary Nodules Less Than 3 Cm in Diameter, With Special Reference to the CT Images. Lung Cancer (2004) 45(1):19–27. doi: 10.1016/j.lungcan.2004.01.009 15196730

[13] Tripathi M. Analysis of Convolutional Neural Network Based Image Classification Techniques. J Innovative Image Processing (2021) 3(2):100–17. doi: 10.36548/jiip.2021.2.003

[14] Desai M Shah M. An Anatomization on Breast Cancer Detection and Diagnosis Employing Multi-Layer Perceptron Neural Network (MLP) and Convolutional Neural Network (CNN). Clinical eHealth (2021) 4:1–11. doi: 10.1016/j.ceh.2020.11.002

[15] Yadav SS Jadhav S. Deep Convolutional Neural Network Based Medical Image Classification for Disease Diagnosis. J Big Data (2019) 6(1):1–18. doi: 10.1186/s40537-019-0276-2

[16] Tian P He B Mu W Liu K Li WJT. Assessing PD-L1 Expression in non-Small Cell Lung Cancer and Predicting Responses to Immune Checkpoint Inhibitors Using Deep Learning on Computed Tomography Images. Theranostics (2021) 11(5):2098–107. doi: 10.7150/thno.48027 PMC7797686 33500713

[17] Wang X Li Q Cai J Wang W Xu P Zhang Y. Predicting the Invasiveness of Lung Adenocarcinomas Appearing as Ground-Glass Nodule on CT Scan Using Multi-Task Learning and Deep Radiomics. Transl Lung Cancer Res (2020) 9(4):1397–406. doi: 10.21037/tlcr-20-370 PMC7481614 32953512

[18] Trebeschi S Bodalal Z Boellaard TN Bucho TMT Beets-Tan RGH. Prognostic Value of Deep Learning-Mediated Treatment Monitoring in Lung Cancer Patients Receiving Immunotherapy. Front Oncol (2021) 11:609054. doi: 10.3389/fonc.2021.609054 33738253 PMC7962549

[19] Shin HC Roth HR Gao M Lu L Xu Z Nogues I. Deep Convolutional Neural Networks for Computer-Aided Detection: CNN Architectures, Dataset Characteristics and Transfer Learning. IEEE Trans Med Imaging (2016) 35(5):1285–98. doi: 10.1109/TMI.2016.2528162 PMC4890616 26886976

[20] Li J Xia T Yang X Dong X Liang J Zhong N. Malignant Solitary Pulmonary Nodules: Assessment of Mass Growth Rate and Doubling Time at Follow-Up CT. J Thorac Dis (2018) 10(Suppl 7):S797–806. doi: 10.21037/jtd.2018.04.25 PMC5945695 29780626

[21] Li W Cao P Zhao D Wang J. Pulmonary Nodule Classification With Deep Convolutional Neural Networks on Computed Tomography Images. Comput Math Methods Med (2016) 2016:6215085. doi: 10.1155/2016/6215085 28070212 PMC5192289

[22] Wang X Zhao X Li Q Xia W Peng Z Zhang R. Can Peritumoral Radiomics Increase the Efficiency of the Prediction for Lymph Node Metastasis in Clinical Stage T1 Lung Adenocarcinoma on CT? Eur Radiol (2019) 29(11):6049–58. doi: 10.1007/s00330-019-06084-0 30887209

[23] Lantuejoul S Rouquette I Brambilla E Travis WD. [New WHO Classification of Lung Adenocarcinoma and Preneoplasia]. Ann Pathol (2016) 36(1):5–14. doi: 10.1016/j.annpat.2015.11.010 26791238

[24] Hasan N Bao Y Shawon A Huang YJSCS. DenseNet Convolutional Neural Networks Application for Predicting COVID-19 Using CT Image. SN Comput Sci (2021) 2(5):1–11. doi: 10.1007/s42979-021-00782-7 PMC8300985 34337432

[25] Pedregosa F Varoquaux G Gramfort A Michel V Thirion B Grisel O. Scikit-Learn: Machine Learning in Python. Mach Learn Res (2011) 12: 2825–30. doi: 10.48550/arXiv.1201.0490

[26] Gould MK Fletcher J Iannettoni M Lynch WR Midthun DE Naidich DP. Evaluation of Patients With Pulmonary Nodules: When Is It Lung Cancer?: ACCP Evidence-Based Clinical Practice Guidelines (2nd Edition). Chest (2007) 132(3):108S–30S. doi: 10.1378/chest.07-1353 17873164

[27] Wahidi M Govert J Goudar R Gould M McCrory D. Evidence for the Treatment of Patients With Pulmonary Nodules: When is it Lung Cancer?: ACCP Evidence-Based Clinical Practice Guidelines (2nd Edition). Chest (2007) 132:94S–107S. doi: 10.1378/chest.07-1352 17873163

[28] McWilliams A Tammemagi MC Mayo JR Roberts H Liu G Soghrati K. Probability of Cancer in Pulmonary Nodules Detected on First Screening CT. N Engl J Med (2013) 369(10):910–9. doi: 10.1056/NEJMoa1214726 PMC3951177 24004118

[29] van Iersel CA de Koning HJ Draisma G Mali WP Scholten ET Nackaerts K. Risk-Based Selection From the General Population in a Screening Trial: Selection Criteria, Recruitment and Power for the Dutch-Belgian Randomised Lung Cancer Multi-Slice CT Screening Trial (NELSON). Int J Cancer (2007) 120(4):868–74. doi: 10.1002/ijc.22134 17131307

[30] Niu R Shao X Shao X Jiang Z Wang J Wang Y. Establishment and Verification of a Prediction Model Based on Clinical Characteristics and Positron Emission Tomography/Computed Tomography (PET/CT) Parameters for Distinguishing Malignant From Benign Ground-Glass Nodules. Quant Imaging Med Surg (2021) 11(5):1710–22. doi: 10.21037/qims-20-840 PMC8047343 33936959

[31] Jemal A Miller KD Ma J Siegel RL Fedewa SA Islami F. Higher Lung Cancer Incidence in Young Women Than Young Men in the United States. N Engl J Med (2018) 378(21):1999–2009. doi: 10.1056/NEJMoa1715907 29791813 PMC7717174

[32] Marcus MW Duffy SW Devaraj A Green BA Oudkerk M Baldwin D. Probability of Cancer in Lung Nodules Using Sequential Volumetric Screening Up to 12 Months: The UKLS Trial. Thorax (2019) 74(8):761–7. doi: 10.1136/thoraxjnl-2018-212263 31028232

[33] Li WJ Lv FJ Tan Y Fu B Chu Z. Benign and malignant pulmonary part-solid nodules: differentiation via thin-section computed tomography. Quant Imaging Med Surg (2022) 12(1):699. doi: 10.21037/qims-21-145 34993112 PMC8666726

[34] Jiang B Zhang Y Zhang L HdB G Vliegenthart R Xie X. Human-Recognizable CT Image Features of Subsolid Lung Nodules Associated With Diagnosis and Classification by Convolutional Neural Networks. Eur Radiol (2021) 31(10):7303–15. doi: 10.1007/s00330-021-07901-1 33847813

[35] Hu X Ye W Li Z Chen C Cheng S Lv X. Non-Invasive Evaluation for Benign and Malignant Subcentimeter Pulmonary Ground-Glass Nodules (</=1 Cm) Based on CT Texture Analysis. Br J Radiol (1114) 2020:93,20190762. doi: 10.1259/bjr.20190762 PMC7548366 32686958

[36] Hu X Gong J Zhou W Li H Wang S Wei M. Computer-Aided Diagnosis of Ground Glass Pulmonary Nodule by Fusing Deep Learning and Radiomics Features. Phys Med Biol (2021) 66(6):065015. doi: 10.1088/1361-6560/abe735 33596552

[37] Liu S Tian G Xu YJN. A Novel Scene Classification Model Combining ResNet Based Transfer Learning and Data Augmentation With a Filter. Neurocomputing (2019) 338:191–206. doi: 10.48550/arXiv.1201.0490

[38] Alzubaidi LS Fadhel MA Al-Shamma O Zhang J Oleiwi SRJAS. Towards a Better Understanding of Transfer Learning for Medical Imaging: A Case Study. Appl Sci (2020) 10:4523. doi: 10.3390/app10134523

[39] Khan SU Islam N Jan Z Din IU Rodrigues JJPRL. A Novel Deep Learning Based Framework for the Detection and Classification of Breast Cancer Using Transfer Learning. Pattern Recognit Lett (2019) 125:1–6. doi: 10.1016/j.patrec.2019.03.022

[40] Liang G Zheng LJCM. A Transfer Learning Method With Deep Residual Network for Pediatric Pneumonia Diagnosis. Biomed Pi (2020) 187:104964. doi: 10.1016/j.cmpb.2019.06.023 31262537

